# Celiac Disease Masquerading as Extreme Thrombocytosis and Severe Anemia in a 52-Year-Old Female Patient: A Rare Case Presentation and Literature Review

**DOI:** 10.7759/cureus.41416

**Published:** 2023-07-05

**Authors:** Cuauhtemoc Jeffrey Soto, Lokeshwar Raaju Addi Palle, Mefthe Berhanu, Yordanos G Negassi, Saima Batool, Shaniah S Holder

**Affiliations:** 1 Internal Medicine, California Institute of Behavioral Neurosciences & Psychology, Fairfield, USA; 2 Surgery, Kamala Children's Hospital, Chennai, IND; 3 Health Sciences, University of Texas Health Science Center at Houston, Houston, USA; 4 Internal Medicine, Learn and Live Wholestic Health Services Clinic, Alexandria, USA; 5 Internal Medicine, Hameed Latif Hospital, Lahore, PAK; 6 Medicine, American University of Barbados School of Medicine, Bridgetown, BRB

**Keywords:** celiac disease, thrombocytosis, anemia, gluten-free diet, atypical presentation, severe anaemia, extreme thrombocytosis, coeliac disease

## Abstract

Celiac disease (CD) is a chronic autoimmune disorder characterized by an immune-mediated response to gluten, resulting in small intestinal mucosal damage. While gastrointestinal (GI) symptoms are commonly associated with CD, atypical presentations can pose diagnostic challenges, particularly when hematological abnormalities are the primary manifestation. We report a case of a 52-year-old female patient who presented with paraesthesia, numbness in her hands and feet, marked thinness, extreme thrombocytosis, severe anemia, and mild electrolyte imbalance. Physical examination was unremarkable, except for the notable thinness. GI symptoms were absent, and there was no family history of gastroenterological diseases. Diagnostic evaluations, including serological tests and duodenal biopsy, confirmed the diagnosis of CD with grade 4 Marsh 3C classification. This case emphasizes the significance of considering CD as a potential cause for atypical hematological manifestations, such as extreme thrombocytosis secondary to severe anemia. Prompt recognition and appropriate management, including adherence to a gluten-free diet, can lead to symptom improvement and resolution of hematological abnormalities. It is crucial for healthcare professionals to recognize and be familiar with these atypical presentations to promote early diagnosis and enhance patient outcomes.

## Introduction

Celiac disease (CD) is a systemic autoimmune disorder that occurs in genetically predisposed individuals, wherein gluten ingestion leads to small bowel injury [1]. Gluten, commonly found in wheat, rye, and barley, is prevalent in various food items such as bread, pastries, cereals, and pasta [2]. The condition affects approximately one in 100 people, with an estimated 3 million Americans being affected, yet only 30% of patients receive a proper diagnosis [1]. Genetic markers for CD, including human leukocyte antigen (HLA) DQ2 and DQ8, are found in affected individuals [3], with HLA-DQ2 being more common, observed in 90% of patients, and HLA-DQ8 being present in the remaining individuals [3].

While CD predominantly occurs in children, it can also arise in adolescents and adults, with delayed introduction of wheat into the diet contributing to its incidence [2]. Patients commonly present with gastrointestinal (GI) features, including bloating, chronic diarrhea, nausea, vomiting, abdominal pain, failure to thrive, delayed puberty, weight loss, anemia, dermatitis herpetiformis, and fat-soluble vitamin malabsorption [3]. CD patients are at an increased risk of developing other autoimmune disorders, such as hypothyroidism, juvenile idiopathic arthritis, and autoimmune hepatitis [1]. In adults, CD manifests systemically, often involving extraintestinal features affecting the liver, bones, reproductive organs, hematologic system, brain, and heart [2].

Atypical CD cases are frequently misdiagnosed due to unawareness of their symptoms. Although the prevalence of atypical cases remains unknown, Admou et al. suggested that approximately 50% of patients may present atypically [4]. Atypical symptoms encompass chronic fatigue, recurrent aphthous ulcers, cerebellar ataxia, epilepsy, psychiatric disorders, recurrent abortions, infertility, and laboratory findings such as transaminitis, vitamin B12 and/or folate deficiency, thrombocytosis, leukocytosis, hypocalcemia, and hypoalbuminemia [4]. Untreated atypical CD can result in complications, including early onset osteopenia or osteoporosis, liver failure, gall bladder dysfunction, peripheral neuropathy, and small bowel cancer [1].

We present a case of a 52-year-old woman presenting with chronic fatigue, dyspnea on exertion, peripheral neuropathy, and weight loss. Laboratory tests revealed iron deficiency anemia, thrombocytosis, and mild transaminitis. Serologic testing and a small bowel biopsy confirmed the diagnosis of CD, which was managed successfully with a gluten-free diet, leading to the complete resolution of symptoms.

## Case presentation

A 52-year-old post-menopausal female patient presented to the clinic with a six-month history of paraesthesia and numbness in her hands and feet, along with fatigue, weakness, and dyspnea on exertion. The patient had no previous history of GI problems, including diarrhea and constipation, and there was no family history of gastroenterological diseases. The patient denied recent travel history and confirmed no use of antibiotics or medications. Over the past six months, she experienced an unintentional weight loss of 3 kg, which corresponds to approximately 5.5% of her initial body weight. Her appetite remained normal, and there were no notable changes in her mood.

On physical examination, the patient appeared pallor and malnourished (based on Mini Nutritional Assessment (MNA), where she received a score of 17), and no tenderness was elicited on abdominal palpation without any palpable masses or organomegaly. She appeared thin, and there were no macroscopic signs of bleeding from the GI or genitourinary tracts. Vital signs were as follows: blood pressure was 110/70 mmHg, and heart rate was increased to 103 beats per minute with a normal body temperature.

Initial investigations included a complete blood count (CBC) with peripheral blood smear, liver function tests (LFTs), renal function tests (RFTs), electrolyte levels, thyroid function tests (TFT), erythrocyte sedimentation rate (ESR), C-reactive protein (CRP), antinuclear antibody (ANA), and rheumatoid factor (RF). The patient's CBC revealed severe anemia, indicated by a hemoglobin (Hb) level of 7.8 g/dL (reference range: 12-15 g/dL), along with extreme thrombocytosis, with a platelet count of 950,000/mm³ (reference range: 150,000-400,000/mm³). The blood indices and the peripheral blood picture, which revealed anisocytosis, hypochromia, microcytosis, and poikilocytosis, further confirmed the presence of iron deficiency anemia (IDA). Table 1 shows all these findings along with the iron studies. Additionally, mild electrolyte imbalances were observed, characterized by hyponatremia and borderline potassium levels. LFTs showed a slight elevation in transaminases, with alanine transaminase and aspartate transaminase values of 57 U/L and 60 U/L, respectively (normal range: 5-35 U/L for both parameters). The renal function tests were found to be within the normal range.

**Table 1:** Lab parameters of the patient.

Laboratory test	Parameter	Result	Normal range
Complete blood count with red blood cell indices	Hemoglobin	7.8 g/dL	12.1-15.1 g/dL
	Mean corpuscular volume (MCV)	66 fl	80-100 fl
	Mean corpuscular hemoglobin concentration (MCHC)	32 g/dL	32-36 g/dL
	Red blood cells	4.2 million cells/µL	4.2-5.4 million cells/µL
	Reticulocyte count	0.6%	0.5-2.5%
	Red cell distribution width (RDW)	15%	11.80-14.80%
	White blood cell count	7.1 cells/mm³	4.5-11 cells/mm³
	Platelet count	950 cells/mm³	150-400 cells/mm³
Iron studies	Iron	30 µg/dL	50-170 µg/dL
	Transferrin	260 mg/dL	200-360 mg/dL
	Transferrin saturation	7.32%	15-50%
	Total iron-binding capacity (TIBC)	410 mcg/dL	300 to 360 mcg/dL
	Ferritin	10 ng/mL	15-150 ng/mL
Serum B12	B12	330	160-950 pg/ml
Vitamin D	Vitamin D level	39 nmol/L	>50 nmol/L
Liver function tests	Alanine aminotransferase (ALT)	57 U/L	7-55 U/L
	Aspartate aminotransferase (AST)	60 U/L	8-48 U/L
	Alkaline phosphatase	180 U/L	44-147 U/L
	Total bilirubin	0.9 mg/dL	0.8-1.2 mg/dL
	Albumin	3.9 mg/dL	3.4-5.4 g/dL
	Total protein	69 g/dL	60-80 g/dL
Renal function tests	Blood urea nitrogen (BUN)	20 mg/dL	6-24 mg/dL
	Creatinine	1.0 mg/dL	0.7-1.3 mg/dL
	Estimated glomerular filtration rate	100 mL/min/1.73m²	>60 mL/min/1.73m²
Electrolytes	Sodium (Na)	133 mmol/L	135-145 mmol/L
	Potassium (K)	3.5 mmol/L	3.5-5.0 mmol/L
	Calcium (Ca)	8.8 mg/dl	8.6-10.3 mg/dl
Thyroid function tests	Thyroid-stimulating hormone (TSH)	2.6 µIU/mL	0.4-4.0 µIU/mL
	Free T4	1.9 ng/dL	0.9-2.3 ng/dL
Inflammatory markers	C-reactive protein (CRP)	10 mg/L	<10 mg/L
	Erythrocyte sedimentation rate	15 mm/hour	0-20 mm/hour
Immune markers	Antinuclear antibody (ANA)	-ve	-ve
	Rheumatoid factor (RF)	-ve	-ve

**Figure 1:**
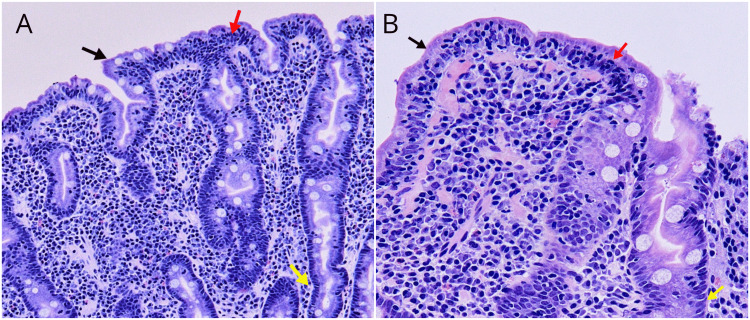
Histological section of the patient's duodenum consistent with celiac disease, Marsh 3C. Hematoxylin and eosin stain showing severe villous atrophy (black arrows), intraepithelial lymphocytes (red arrows), and crypt hyperplasia (yellow arrows). A: 10x zoom. B: 40x zoom.

The patient was subsequently admitted to the internal medicine ward to initiate comprehensive management and treatment. With the aim of evaluating the patient's symptoms and laboratory abnormalities in greater detail, further investigations were undertaken to find out the cause of her IDA. Abdominal ultrasound was performed and showed normal findings with no hepatosplenomegaly. Bone marrow aspiration and biopsy showed no evidence of myeloproliferative disorders or neoplastic conditions. Stool samples were tested for occult blood but they were negative. However, screening tests for CD revealed elevated levels of immunoglobulin A (IgA), anti-tissue transglutaminase (anti-tTG) antibodies, and anti-endomysial antibodies (EMA). To confirm the diagnosis, a duodenal biopsy was performed. In the histopathological examination, the duodenal biopsy revealed marked villous blunting, atrophy, crypt branching, an increase in intraepithelial lymphocytes, moderate chronic lymphoplasmacytic infiltration in the lamina propria, and focal Brunner's gland hyperplasia. These findings are suggestive of grade 4 Marsh 3C classification, indicating severe mucosal damage and confirming the diagnosis of CD (Figure 1).

The initial treatment plan involved initiating a strict gluten-free diet, which included educating the patient about foods containing gluten to be avoided, providing guidance on gluten-free alternatives, and recommending multivitamin supplements. Blood transfusion and intravenous (IV) iron supplementation (ferric derisomaltose 1000 mg as a single dose) were also administered to address the severe anemia. Close monitoring of electrolyte levels and correction of any imbalances were implemented. The patient was prescribed 325 mg of oral ferrous sulfate, which provides 65 mg of elemental iron, to be taken every other day for a duration of six months.

Following discharge, the patient was scheduled for regular follow-up visits at monthly intervals for the first six months. During these visits, clinical assessment, monitoring of Hb levels, platelet counts, electrolyte levels, and any new symptoms were performed. Nutritional counseling and adherence to the gluten-free diet were reinforced during these visits.

At the six-month follow-up, the patient reported a significant improvement in symptoms, including a resolution of paraesthesia and numbness in the hands and feet. Laboratory investigations demonstrated a gradual normalization of Hb levels, platelet counts, and electrolyte levels within the reference range. The patient continued to adhere to the strict gluten-free diet, and no further complications or relapses were observed.

## Discussion

Persons with a high risk of CD include first-degree relatives of patients with CD, persons with CD-associated symptoms (diarrhea and abdominal pain), and persons with CD-associated disorders such as diabetes mellitus type 1, osteoporosis, and anemia [5]. The prevalence of CD in these patients is 4.5% [5]. The pathophysiology of CD involves the inappropriate activation of T-lymphocytes against gluten-derived proteins (gliadins and glutenins) [6]. Gliadin interacts with the small bowel enterocytes and disassembles the inter-enterocyte tight junctions leading to increased gut permeability [6]. These peptides then pass the epithelial barrier and enter the lamina propria where they activate CD4+ T cells [6]. T-lymphocytes stimulate the release of proinflammatory cytokines and cytotoxic T-cells and activate T-helper 2 cells, which lead to plasma cell and antibody secretion. Anti-gliadin, anti-EMA, and anti-tTG antibodies are commonly found in serology in persons with CD [6]. This is responsible for the GI symptoms seen in classic CD; however, many patients have subtle symptoms affecting the various organ systems.

The most common hematologic manifestation of atypical CD is anemia, which is found in 12-69% of cases and is more common in adults than children [1]. Anemia is secondary to iron, folate, or vitamin B12 deficiency [7]. Iron deficiency is the most common subtype and is caused by impaired absorption of iron in the duodenum; in some cases, occult blood loss from the GI tract may be the cause of IDA [7]. Features of IDA include fatigue, conjunctival pallor, generalized weakness, tachycardia, and shortness of breath or chest pain on exertion, all of which were present in this patient [8]. IDA is characterized by microcytic, hypochromic red blood cells on peripheral smears [8]. Complete blood count and iron studies will show low hemoglobin and hematocrit, decreased mean corpuscular volume, low serum iron, elevated total iron-binding capacity, and low ferritin levels [8]. Thrombocytosis may be due to hyposplenism or secondary to IDA [7]. Asymptomatic reactive thrombocytosis occurs in 60% of CD cases and is due to increased megakaryocyte proliferation in the setting of IDA [9]. Other hematologic findings in CD include thrombocytopenia, leukocytosis or leukopenia, and immunoglobulin A (IgA) deficiency. In this case, the patient had symptomatic anemia with a hemoglobin count of 7.8 g/dl and severe thrombocytosis with a count of 950,000/mm³.

In CD, peripheral neuropathy, characterized by paresthesia, pain, numbness, and weakness in the limbs, may be a result of vitamin B12 deficiency [10]. The most common subtypes of peripheral neuropathy reported in CD are sensory axonal and small fiber sensory polyneuropathies [10]. Our patient had a six-month history of paresthesia and numbness in both of her hands and feet. CD can lead to electrolyte disturbances, which can result in arrhythmias, altered mental status, and death. This can be seen in the celiac crisis, which is a rare but serious complication of CD and is caused by severe metabolic disturbances [11]. Electrolyte changes found on laboratory tests include hyper/hyponatremia, hypokalemia, hypomagnesemia, and hypocalcemia [11]. Symptoms include profuse diarrhea and signs of severe dehydration, neurologic dysfunction, renal dysfunction with a creatinine level of more than 2.0 g/dL, and more than 10 lbs weight loss [11]. Although the patient, in this case, did not have a celiac crisis, there were mild electrolyte abnormalities with mild hyponatremia and borderline normal potassium.

Table 2 summarizes various reports of atypical CD with hematologic manifestations, highlighting the patients’ presentation, type of hematologic abnormality, significant diagnostic findings, and the treatment and prognosis [12-15]. In addition to the cases mentioned, our case aims to increase physician awareness about the association between CD and hematologic manifestations, specifically anemia and thrombocytosis, which may be the only features present in some cases.

**Table 2:** Literature review exploring cases of CD with hematologic manifestations. CD: celiac disease; IDA: iron deficiency anemia; plt: platelet; N/S: not specified; Hb: hemoglobin; N: number of patients.

Author, year	Patient sample	Symptoms	Hematologic manifestations	Serology	Biopsy findings	Treatment and prognosis
Carroccio et al. (2002) [12]	83-year-old female	Chronic fatigue only	IDA (Hb: 4 g/dl), extreme thrombocytosis (plt count: 1,400,000/mm3), hyposplenism	+ Anti-endomysial IgA antibodies, + anti-transglutaminase IgA antibodies	Classic findings of CD with total villous atrophy (Marsh classification IIIC)	Gluten-free diet only, which led to normalization of Hb and platelet count. One year later, intestinal biopsies showed normal villi and crypts without inflammatory infiltration
Corazza et al. (1995) [13]	200 patients	Asymptomatic	Anemia only	+ Antigliadin antibody (N = 16), + anti-endomysial antibody (N = 10)	Jejunal biopsy findings consistent with celiac disease (N = 10)	N/S
Voigt et al. (2008) [14]	25-year-old female	Vertigo fatigue	IDA (Hb: 4.6), severe thrombocytosis (plt count: 1,700,000), vitamin B12 def (112; N = 133-675)	Not taken	Classic findings of CD with partial villous atrophy (Marsh classification 1)	Blood transfusion, which improved Hb and platelet count. Long-term gluten-free diet and iron supplementation, which led to symptom resolution and no recurrence of symptoms
Unsworth et al. (2000) [15]	483 patients	Asymptomatic	IDA	IgA anti-endomysial antibodies (N = 32)	25 agreed for a biopsy and 22 out of 25 had typical findings of CD	N/S

The diagnosis of CD with atypical features may be missed since symptoms may overlap with more prevalent conditions. Other conditions that cause anemia and thrombocytosis such as myeloproliferative disorders, infectious diseases, metastatic cancer, and other autoimmune diseases may cause the diagnosis of CD to be overlooked [9]. Therefore, a high index of clinical suspicion is required to diagnose CD, especially in atypical cases with hematologic presentations only. Serologic tests and small bowel biopsy (SBB) aid in confirming the diagnosis of CD. The presence of EMA and tTG antibodies on laboratory tests is highly sensitive and specific for CD. A study by Niveloni et al. found that immunoglobulin G (IgG) anti-deamidated gliadin-related peptide (a-DGP) had a diagnostic accuracy that was equivalent to established assays and could detect CD in IgA-competent and IgA-deficient patients [16]. Although positive serology findings are a good indicator for biopsy, some patients may be seronegative. The presence of CD antibodies correlates with the severity of villous atrophy; however, patients with a lesser degree of atrophy can be seronegative [4]. In these cases, HLA typing must be done and if positive, then a duodenal biopsy should be performed [4].

Endoscopic biopsy of the duodenal mucosa is the gold standard diagnostic test for CD detection. Characteristic histopathological findings include (i) villous atrophy, (ii) crypt hyperplasia, and (iii) increased intraepithelial T lymphocytes [6]. A study by Veress et al. aimed to discover the normal number of intraepithelial lymphocytes in the human small bowel [17]. They concluded that values between 25 and 29 lymphocytes per 100 enterocytes were registered as "borderline" and above 30 per 100 enterocytes was pathologic intraepithelial lymphocytosis [17]. The Marsh classification system was introduced in 1992 and describes the stages of small intestine damage found on histology in persons with CD. Table 3 shows the histologic findings in Marsh classification.

**Table 3:** Marsh classification in the evaluation of histopathological findings in celiac disease. Marsh criteria A, B, and C also include intraepithelial lymphocytosis and crypt hyperplasia but are differentiated based on the degree of villous atrophy [6].

Marsh classification	
Marsh 0	Normal mucosal architecture without significant intraepithelial lymphocytic infiltration
Marsh I	Lymphocytic enteritis: normal mucosal architecture with marked infiltration of villous epithelium by lymphocyte (>30 lymphocytes per 100 enterocytes)
Marsh II	Lymphocytic enteritis with crypt hyperplasia
Marsh III	Intraepithelial lymphocytosis, crypt hyperplasia, and villous atrophy
Marsh IIIA	Partial villous atrophy: villi blunt and shortened
Marsh IIIB	Subtotal villous atrophy
Marsh IIIC	Total villous atrophy, mucosa resembles colonic mucosa

In the cases of patchy mucosal involvement, or mild symptoms, SBB may yield false-negative results [2]. Villous atrophy may be most severe in the proximal jejunum, which is not typically reached by endoscopic biopsy in some cases of CD [2]. Due to this, serologic testing, SBB, and, in controversial cases, HLA typing are required to confirm the diagnosis of CD in atypical cases [2].

A lifelong gluten-free diet is the main mode of therapy that leads to the complete resolution of all symptoms [4]. Clinical improvement occurs weeks after dietary changes, and mucosal regeneration occurs within one to two years [4]. Alternative modes of treatment under investigation include supplemental enzyme therapy with microbial prolyl-endopeptidase, which has been found to accelerate gluten digestion in the GI tract leading to decreased interaction between the enterocytes and gluten-derived peptides [18]. In patients with atypical CD with severe anemia, iron supplementation with ferrous sulfate is required to resolve the physical symptoms and resolve the reactive thrombocytosis [19]. In patients who have duodenal atrophy or the inability to tolerate iron sulfate, then sucrosomial iron can be given. Iron sulfate supplementation is associated with GI side effects such as abdominal pain, nausea, vomiting, bloating, and flatulence. Sucrosomial iron is a formulation in which ferric pyrophosphate is enclosed within a phospholipid bilayer and is absorbed via paracellular and transcellular routes in the small bowel. In a study by Elli et al., the authors compared patients with CD and IDA who were on ferrous sulfate and sucrosomial iron [19]. They discovered that although there was no significant difference in iron parameter improvement, patients on sucrosomial iron had reported a lower severity of GI symptoms and a higher increase in quality of life when compared to the ferrous sulfate group [19]. They concluded that sucrosomial iron is beneficial for patients with CD and IDA and a known intolerance to iron sulfate [19].

Practicing a strict gluten-free diet and close follow-up at regular intervals are important in patients with CD and severe anemia since the complications of anemia can be fatal. During follow-up visits, hemoglobin, platelet, and electrolyte levels should be monitored until stabilized, and nutritional counseling about the importance of a gluten-free diet should be given to ensure patient adherence. These were implemented in the patient and led to the complete resolution of her anemia and associated symptoms, thrombocytosis, and peripheral neuropathy with no further complaints.

## Conclusions

CD affects a significant portion of the population, particularly children, and typically manifests with GI symptoms. However, adults often present with extraintestinal manifestations that impact multiple organ systems. Atypical CD, characterized by severe hematologic manifestations, is infrequently observed and commonly underdiagnosed due to the prominence of more prevalent differential diagnoses. It is crucial for physicians to recognize that not all cases of CD exhibit intestinal symptoms, and in patients with anemia, conducting a CD serologic panel with or without an SBB is essential to confirm or exclude the diagnosis. Implementing a lifelong strict gluten-free diet and incorporating supplemental iron intake is imperative for patients with CD and hematologic manifestations, as it can lead to symptom resolution and prevent complications. Further research is warranted to explore additional diagnostic and therapeutic strategies, enabling early diagnosis and optimal treatment approaches for CD with hematologic manifestations, ultimately enhancing patients' quality of life.
